# Emergency care capacity in Freetown, Sierra Leone: a service evaluation

**DOI:** 10.1186/s12873-015-0027-4

**Published:** 2015-02-03

**Authors:** Rachel M Coyle, Hooi-Ling Harrison

**Affiliations:** Department of Primary Care and Public Health, King’s College, London, England SE1 3QD UK; King’s Sierra Leone Partnership, King’s Centre for Global Health, Weston Education Centre, London, England SE5 9RJ UK

**Keywords:** Emergency medicine, Emergency medical systems, Low-income country

## Abstract

**Background:**

There is an increasing global recognition of the role of emergency medical services in improving population health. Emergency medical services remain underdeveloped in many low income countries, particularly in sub-Saharan Africa. There have been no previous evaluations of specialist emergency and critical care services in Sierra Leone.

**Methods:**

Emergency care capacity was evaluated at a sample of seven public and private hospitals in Freetown, the capital of Sierra Leone. A structured set of minimum standards necessary to deliver emergency and critical care in the low-income setting was used to evaluate capacity. The key dimensions of capacity evaluated were infrastructure, human resources, drug and equipment availability, training, systems, guidelines and diagnostics. A score for each dimension of capacity was calculated based on the availability of a list of specified indicators within each dimension. In addition, an Emergency Care Capacity Score was calculated to demonstrate a composite measure of capacity based on the various indicator scores. This method has been used by the World Health Organisation in evaluating the availability and readiness of healthcare systems in low- and middle-income countries.

**Results:**

Substantial deficiencies in capacity were demonstrated across the range of indicators and predominantly affecting publically funded facilities. Capacity was weakest in the domain of infrastructure, with an average score of 43%, while the strongest areas of capacity overall were in drug availability, 82%, and human resources, 79%. A marked disparity was noted between public and private healthcare facilities with consistently lower capacity in the former. The overall Emergency Care Capacity Score was 66%.

**Conclusion:**

There are substantial deficiencies in emergency care systems in public hospitals in Freetown which are likely to compromise effective care. This represents a serious barrier to access to emergency healthcare. Emergency care systems have an important role in improving population health and as such should a priority for local policy makers.

**Electronic supplementary material:**

The online version of this article (doi:10.1186/s12873-015-0027-4) contains supplementary material, which is available to authorized users.

## Background

Underdeveloped emergency medical services are associated with poor health outcomes [1]. In recent years there has been increasing recognition that emergency medical services have a role to play in improving population health in low- and middle-income countries (LMIC) [1,2]. The World Health Organisation has urged member states to “assess comprehensively the pre-hospital and emergency-care context including, where necessary, identifying unmet needs” [3]. A structured needs assessment is an essential first step in health service development and is necessary to establish existing capacity and identify priorities for development [4].

The Republic of Sierra Leone is a small West African country with a population of nearly six million [5]. Ranked 177 out of 182 in the United Nations Human Development Index Sierra Leone is one of the world’s least developed countries in terms of its economic, education and health indices. Life expectancy at birth is just 48 years, substantially below the regional and global averages of 55 and 70 years respectively [5,6], a figure explained by a heavy burden of communicable disease, trauma, and maternal and child disease on a background of poverty, malnutrition and inadequate access to healthcare [7].

Previous studies evaluating surgical and obstetric services in Sierra Leone have demonstrated substantial deficiencies in capacity across a range of areas [8,9]. It is highly unlikely that these deficiencies are limited solely to the fields of surgery and obstetrics but rather are transferable across the healthcare system. As such an evaluation of emergency care capacity in Sierra Leone is timely.

The King’s Sierra Leone Partnership (KSLP) was established in 2011 with the aim of improving health outcomes in Sierra Leone by working in partnership with key healthcare stakeholders in Sierra Leone to increase health system capacity [10]. Strengthening emergency care delivery is a core pillar of the work of KSLP in Sierra Leone. A structured needs assessment was undertaken to establish existing capacity to deliver emergency medical care in Freetown, Sierra Leone.

## Methods

An assessment of capacity to deliver emergency and critical care was carried out in a sample of secondary care facilities in Freetown using a structured set of standards developed by Baker et al. to evaluate emergency and critical care capacity in Tanzania [11]. This survey tool outlines the first published guidelines for emergency and critical care system requirements in LIC. Although this tool has not been validated, its key strength lies in the process of its development, which involved both a review of evidence based international guidelines for trauma, surgery, anaesthesia, and paediatrics; and obtaining expert opinions from clinicians with experience of working in LIC [11]. The tool clearly outlines the minimum requirements for effective emergency and critical care delivery which are both relevant and realistic in LIC. Capacity in the areas of infrastructure, human resources, training, drugs, equipment, systems, guidelines and diagnostics was evaluated as part of a structured needs assessment for emergency care facilities in Freetown. A full list of the indicators within each area of capacity is available in Additional file 1.

There are nine government hospitals in Freetown which provide general or specialist, (paediatric or obstetric), emergency care. There are six district facilities which include two police/military facilities, and three tertiary facilities. In addition, there are five private hospitals which provide emergency care, three private reproductive facilities and one private eye hospital, for the purpose of the study ‘private’ refers to any non-government funded hospital, including non-governmental organisation (NGO) hospitals, mission hospital, and private-for-profit facilities [12]. A convenience sample of seven hospitals was selected which included two tertiary government facilities, three district government facilities and two general private facilities, police/military facilities were excluded.

Through inclusion of both tertiary and district government hospitals the sample is felt to be representative of emergency care services available in Freetown. Two private facilities were also included in the evaluation to investigate any difference in capacity between these facilities and government hospitals.

Seven secondary care facilities, two private and five public, were invited to participate in the study. All seven facilities consented to participation. All data was collected by the primary researcher, (RC), using the data collection tool described above. Hospital visits lasted between one and two hours. The survey was completed with either the hospital’s lead clinician, (n = 4), or a senior member of medical staff appointed by the lead clinician, (n = 3). Data was stored and analysed in Excel. There were no missing data.

Results are presented as the proportion of facilities which had each indicator within the various domain of capacity. For example if all seven facilities answered positively in respect to a given component of capacity, the score for that indicator would be 100% (7/7) while if only facility answered positively the score would be 14%, (1/7). The average score per domain is presented as a composite measure of capacity in that area. An overall Emergency Care Capacity Score (ECCS), a measure designed to incorporate the information on each domain of capacity, was calculated as an average of the individual category scores.

One paediatric facility was included in the evaluation. Consequently this facility was excluded from calculations pertaining to adult emergency care services only. As such n = 7 for components of capacity relating to adult and paediatric services, and n = 6 for those applicable only to adults, as is indicated in the text and figures below.

### Ethical considerations

In consultation with King’s College London Department of Primary Care and Public Health it was agreed that the type of research undertaken falls into the category of service evaluation, and as such is exempt from ethical board review. However, the project was designed because the need for comprehensive evaluation of existing emergency care services was highlighted by both local and international stakeholders in the King’s Sierra Leone Partnership. Written consent was obtained from the lead clinician in each participating hospital. Information identifying individual hospitals has been removed.

## Results

A full list of capacity indicators is available in Additional file 1. Capacity was lowest in the domains of infrastructure and guidelines and strongest in human resources and drug availability. The Emergency Care Capacity Score (ECCS), a composite measure of capacity based on performance in the various domains, was 66% for all facilities.

### Infrastructure

The two private facilities included in the evaluation routinely triaged adult and paediatric patients according to medical need. In contrast, no public facility had triaging systems in place for adult patients and two public facilities had paediatric triage. Two facilities, both private, had an EC with resuscitation facilities for adults. Three facilities, two private and one public, had resuscitation facilities for children. Four of the seven hospitals had an intensive care unit (ICU). None of the district hospitals had an ICU.

**Figure 1:**
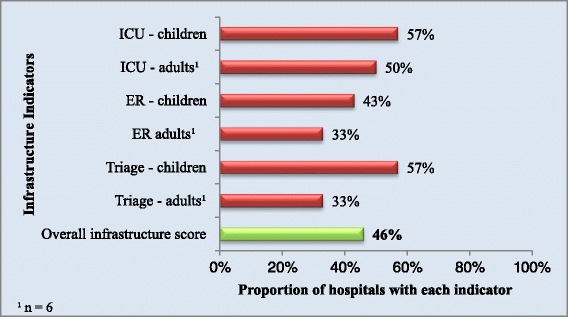
**Proportion of hospitals with each infrastructure indicator and overall infrastructure score.**

The two private facilities assessed had all of the six infrastructure indicators while the five public facilities had an average of one infrastructure indicator. The overall score for infrastructure, based on the various components of capacity described, was 46% and is displayed in Figure 1.

### Human resources

In four of the seven hospitals, 57%, there was a dedicated EC nurse. Although all facilities reported that a clinician was based in the EC during working hours, only four facilities had out-of-hours clinician cover. All hospitals which had an ICU, (n = 4), reported having a medical head of ICU and a higher staff to patient ratio in ICU.

**Figure 2:**
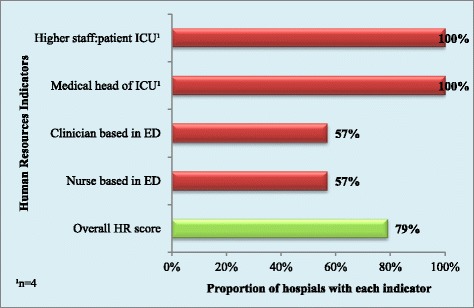
**Proportion of hospitals with each human resources indicator and overall human resources score.**

The two private facilities had all four of the HR indicators while the five public facilities had an average of two HR indicators. The overall HR score was 79% and is displayed in Figure 2.

### Training

The minimum requirement for training was defined as having attended an educational course organised within a government hospital or organisation. It is recognised that the lack of formal postgraduate clinical training in Sierra Leone complicates assessment of the level of an individual’s training. Triage training was reported least frequently with only three hospitals reporting that staff had undergone training in adult triage. Four reported have had training, in paediatric triage. Formal training in adult critical care was reported most frequently, six of the seven hospitals assessed. Importantly, only one hospital organised regular, (biannual), in-house acute care courses to provide staff with regular opportunities to undergo training and to ensure consistency of teaching.

**Figure 3:**
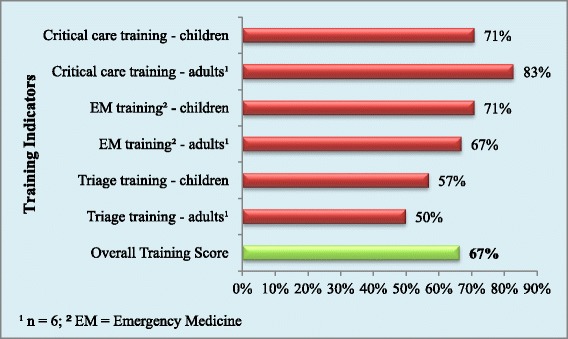
**Proportion of hospitals with each training indicator and overall training score.**

Public facilities had an average of four of the six training indicators while both private facilities reported having all six indicators. The overall score for training capacity was 67% and is shown in Figure 3.

### Drugs and equipment

All facilities stocked intravenous (IV) gentamicin and six of seven facilities stocked IV penicillin and quinine. Oral rehydration solution and IV fluids were stocked in all facilities. Of note, only two facilities (both private) stocked insulin.

All facilities stocked equipment required to carry out IV procedures, such as cannulae and fluid giving sets. Only three facilities had oxygen concentrators or cylinders available in the EC. Light was felt to be unsuitable for clinical examination in four of the seven facilities.

**Figure 4:**
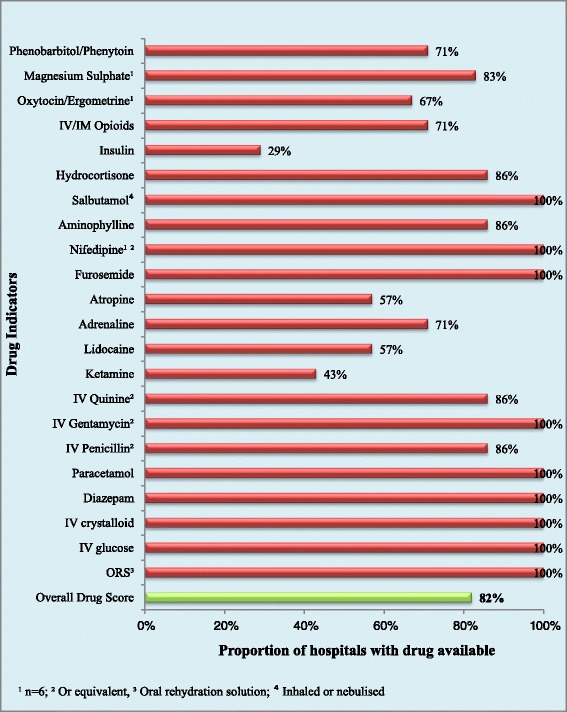
**Proportion of hospitals with each drug indicator and overall drug availability score.**

**Figure 5:**
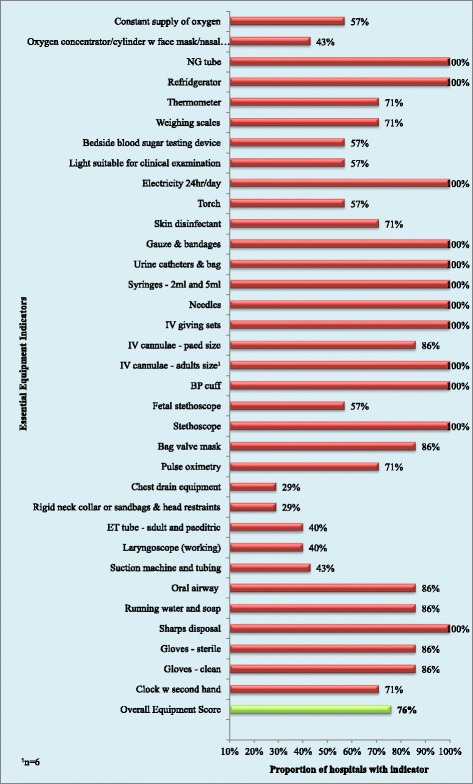
**Proportion of hospitals with each equipment indicator and overall equipment availability score.**

The private hospitals stocked all 23 indicator drugs and all 34 equipment indicators. The public facilities had an average of 16 of the 21 drug indicators and 21 of the 34 equipment indicators. The overall scores for drugs and equipment availability were 82% and 76% respectively and are displayed in Figures 4 and 5.

### Systems

Two hospitals had a system in place for delaying registration and payment until after the receipt of emergency treatment for critically unwell adults, and three had a similar system for children. All facilities reported that vital signs were monitored at least twice daily in acutely unwell adult and paediatric patients. Only two facilities had a system in place to identify ward patients whose clinical condition was deteriorating. The score for systems indicators was 58%. Private facilities had an average of 11 of the 12 systems indicators. The public facilities had an average of 4 of the 12 systems indicators.

### Guidelines

**Figure 6:**
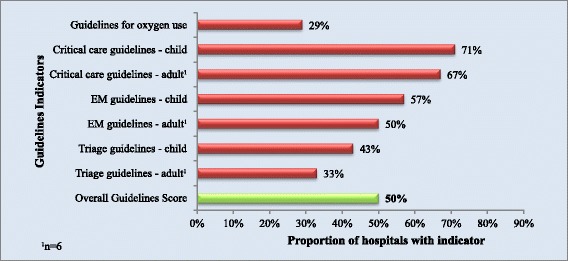
**Proportion of hospitals with each guidelines indicator and overall guidelines score.**

Guidelines for critical care were reported most frequently with five facilities having guidelines for paediatric critical care, (n = 7), and four having guidelines for adult critical care (n = 6). Emergency care guidelines for children were present in four facilities and for adults in three. Paediatric triage guidelines were reported in three facilities, adult triage guidelines were reported in two facilities. Two hospitals reported having guidelines for oxygen therapy. Public facilities had an average of 2 of the 12 guidelines indicators when private facilities had an average of 11. The overall score for guidelines indicators was 50%, and is displayed in Figure 6.

### Diagnostics

All hospitals had facilities to check haemoglobin and blood glucose. Measurement of renal function was available in five facilities. Radiography was available in four facilities although no district hospital had access to x-ray facilities. Emergency blood transfusion was available in the tertiary and private facilities, but not in the district hospitals. The average score for diagnostics was 75%. Private facilities had an average of eight of the nine diagnostics indicators while public facilities had an average of six.

### Emergency care capacity score

**Figure 7:**
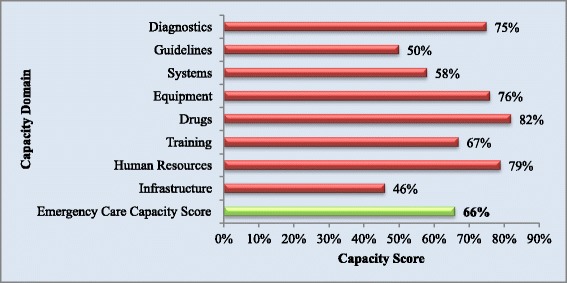
**Overall capacity scores by category and Emergency Care Capacity Score of hospitals with each infrastructure indicator and overall infrastructure score.**

An overall emergency care capacity score, an assessment of the readiness of healthcare services in Freetown to deliver emergency care services, was calculated based on average capacity in each domain. ECCS was 66% for all hospitals when all dimensions of capacity were evaluated. This is illustrated in Figure 7.

A substantial variation in capacity was found between tertiary government, district government and private facilities with consistently lower capacity found in government hospitals. When stratified by facility type the lowest ECCS was found in district hospitals, 36% compared to 64% in tertiary facilities and 99% in private hospitals. When stratified by funding source, government hospitals had an average ECCS of 53% compared to 99% in district hospitals. Results by facility and funding type are detailed in Table 1.

**Table 1:** **Capacity stratified by facility and by funding source**

	**Infrastructure**	**Human resources**	**Training**	**Drugs**	**Equipment**	**Systems**	**Guidelines**	**Diagnostics**	ECCS*
**Facility**									
Tertiary	50%	100%	50%	57%	69%	46%	43%	100%	**64%**
District	6%	0%	56%	77%	63%	28%	19%	44%	**37%**
Private	100%	100%	100%	100%	100%	96%	100%	94%	**99%**
**Funding**									
Government	23%	70%	57%	68%	65%	40%	61%	37%	**53%**
Private	100%	100%	100%	100%	100%	96%	100%	94%	**99%**

*ECCS – Emergency Care Capacity Score. The Emergency Care Capacity Score displays a composite measure of capacity in all 8 domains of capacity.

## Discussion

This study demonstrates widespread deficiencies in capacity in government funded hospitals in Freetown, which is likely to negatively impact on service delivery. Capacity was weakest in infrastructure and guidelines, and highest in drug availability and human resources. A marked disparity was demonstrated between government and private facilities with consistently lower capacity in government facilities.

Weaknesses in emergency care infrastructure have been well described in LMIC [2]. While many hospitals have a designated area in which acutely ill patients are assessed, frequently this lacks essential facilities such as resuscitation equipment, triage or a reliable supply of emergency drugs and equipment [13]. The importance of triage systems in prioritising the expedient treatment of critically unwell patients has been recognised globally [14]. The absence of a triaging system in many hospitals in Freetown prevents the differentiation of the acutely unwell from the clinically stable, creating delays in the identification and treatment of medical emergencies. Limited resuscitation facilities in government hospitals are likely to exacerbate this, as if a patient cannot be resuscitated in the EC due to lack of space or medication he or she will have to be transferred to the ward, creating further time delays between diagnosis and the receipt of treatment [15,16].

Guidelines can be used to improve consistency of clinical care and aid decision making, particularly where healthcare staff have limited emergency care training opportunities and senior support is limited [17]. Disease specific guidelines have been used in resource constrained settings with positive results. For example in Malawi the introduction of a protocol to deal with open fractures resulted in functional outcomes similar to those in high income countries [18]. A significant reduction in mortality from childhood pneumonia was demonstrated in a tertiary hospital in Papua New Guinea, relative risk of death post intervention 0.65 (95% CI 0.52-0.78), following introduction of the routine use pulse oximetry to guide oxygen therapy [19]. Given the limitations in training opportunities and shortages of healthcare workers demonstrated, guidelines may provide a means of improving clinical outcomes in the context of limited resources in Sierra Leone. Guidelines should be targeted to address priorities identified by hospital staff, and by the local disease burden.

Scores in drug and equipment were among the highest. However, although capacity was strong in the domains of drugs and equipment it should be emphasised that in government hospitals and private-for-profit facilities, with the exception of patients covered by the country’s Free Health Care Initiative, (i.e. under-5 s and pregnant and lactating women), user fees are charged for all drugs and equipment. Therefore, although the drugs and equipment required to treat a particular emergency may be present within the hospital pharmacy, user fees may make such items prohibitively expensive for the poorest patients and the expectation of these costs may even preclude seeking healthcare [20].

The survey tool has also been used to evaluate emergency and critical care capacity in Tanzania [11]. As in our study, deficiencies in capacity were most prominent in infrastructure and guidelines, while capacity was higher in terms of drug and equipment availability. In contrast with the Tanzanian study we found higher levels of emergency and critical care training reported in the hospitals assessed. At the time of carrying out the evaluation there was no formal postgraduate training for clinicians in critical or emergency care in Sierra Leone. This complicates truly ascertaining the level of training an individual has had and it may be that there is over-reporting of training. Conversely limited opportunities for postgraduate training lead to some doctors and nurses moving abroad to specialise. Given that the training criteria used in our evaluation are met if any member of staff in the hospital has had specialist training, it may be that one or two trained individuals in a hospital lead to a positive answer with respect to the criteria but in fact mask more significant training deficiencies.

Potential limitations of the survey tool should be recognised. For example, while indicators such as the presence of a dedicated emergency department or a particular medication can be easily verified, evaluating training or clinical activity is more difficult and relies on self-reporting, which may introduce bias. However, a key strength of this tool is in its rigorous development, as discussed above. Moreover the consistency overall of our results not only with the Tanzanian study on which it was based, but also with evaluations of surgical and obstetric capacity in Sierra Leone, lend weight to the validity of our findings [8,9].

The limitation of our study to the Freetown area limits the generalizability of our findings to the country as a whole. It is recognised that there is a mal-distribution of healthcare resources, in terms of facilities and healthcare workers, in Sierra Leone [7]. Moreover, hospitals in the Western Area, in which Freetown in situated, tend to be better equipped than those elsewhere [12]. As such, although we have demonstrated substantial limitations in capacity it is likely that these overestimate the capacity to deliver emergency care in Sierra Leone.

Another limitation is the small number of facilities included in the evaluation, which preclude the use of inferential statistics in the analysis. However the aim of the evaluation was to establish a baseline assessment of capacity to deliver emergency care in Freetown. To this end two of three tertiary government hospitals were included, three of four district government hospitals and two of three private hospitals.

The results of this evaluation have been presented to local stakeholders in the King’s Sierra Leone Partnership and used to highlight development priorities. This needs assessment was carried out to set a baseline at the beginning of a long partnership between KSLP, the Ministry of Health and Sanitation of Sierra Leone and the College of Medical and Allied Health Sciences of Sierra Leone. The immediate focus of KSLP has been improving service delivery at Freetown’s main government hospital, Connaught Hospital. Since the evaluation was carried out a training system based on the South African Triage Score has been adopted at Connaught Hospital and the Ministry of Health and Sanitation has supported the roll-out of this nationally in due course. The triage system is reported to be facilitating care at Connaught Hospital during the current Ebola epidemic in Sierra Leone, however in light of the current epidemic further service development has been postponed.

## Conclusions

Widespread deficiencies demonstrated in government hospitals in Freetown indicate inadequate capacity to deliver effective emergency care in the city. These limitations compromise patient access to high quality emergency care in the city and are likely to contribute to poor morbidity and mortality outcomes in these settings. In light of increasing international recognition of the role of emergency medicine in improving population health in LIC, improving emergency care delivery in government hospitals should be a priority for local policy makers.

## Additional file

Additional file 1:
**Structure Standards for Emergency and Critical Care in Low Income Countries.**

## Abbreviations

CMCD: Communicable, maternal and childhood disease
ECCS: Emergency care capacity score
EC: Emergency centre
EMS: Emergency medical services
ER: Emergency room
HR: Human Resources
ICU: Intensive care unit
IV: Intravenous
KSLP: King’s Sierra Leone Partnership
LIC: Low-income countries
LMIC: Low- and middle-income countries
NCD: Non-communicable disease
NGO: Non-governmental organisation
OPD: Out-patient department
OPP: Out-of-pocket
SATS: South African Triage Score
WHO: World Health Organisation
