# Supplementary material for: Emergency care capacity in Freetown, Sierra Leone: a service evaluation
Source: BMC Emerg Med. 2015 Feb 3;15(1):2. doi: 10.1186/s12873-015-0027-4 (PMC4320466; doi:10.1186/s12873-015-0027-4)
Supplement: Additional file 1: — Structure Standards for Emergency and Critical Care in Low Income Countries. [file 12873_2015_27_MOESM1_ESM.pdf]

Structure Standards for Emergency and Critical Care in Low Income Countries

The hospital should have

An infrastructure designed for managing emergency and critically ill adults and children

**Infrastructure Indicators**	 1<br>2	Designated Triage Area<br>For adults<br>For children
	 3<br>4	Designated Emergency Room / Area<br>For adults<br>For children
	 5<br>6	An ICU or a designated area of a ward for critically ill patients<br>For adults<br>For children

Sufficient human resources for managing emergency and critically ill adults and children

**Human Resource Indicators**	7	Nurse/other health worker either working in the ER or having the task of immediately going to the ER if a critically ill patient arrives
	8	A clinician either working in the ER or being "on-call" for the ER if a critically ill patient arrives
	9	A designated "Medical Head of ICU"
	10	A higher ratio of staff: patients on ICU than on general wards

Sufficiently trained staff for managing emergency and critically ill adults and children

**Training Indicators**	11	Nurses and clinicians involved in adult triage have been trained in adult triage
	12	Nurses and clinicians involved in emergency care of adults have undergone training in emergency care
	13	Nurses and clinicians involved in critical care for adults have undergone training in critical care
	14	Nurses and clinicians involved in paediatric triage have been trained in paediatric triage
	15	Nurses and clinicians involved in emergency care of children have undergone training in emergency care
	16	Nurses and clinicians involved in critical care for children have undergone training in paediatric critical care

Essential drugs for emergency and critical care

**Drug Indicators**	17	Oral Rehydration Solution
	18	IV glucose
	19	IV crystalloid (Normal Saline ± Ringers Lactate)
	20	Diazepam
	21	Paracetamol
	22	Parenteral Penicillin (or equivalent)
	23	Parenteral Gentamycin (or equivalent)
	24	Parenteral Quinine (or other anti-malarial)
	25	Ketamine
	26	Lidocaine
	27	Adrenaline
	28	Atropine
	29	Frusemide
	30	Nifedipine or other anti-hypertensive
	31	Aminophylline
	32	Salbutamol (for inhaler or nebuliser)
	33	Hydrocortisone
	34	Insulin
	35	IV/IM opioids
	36	Naloxone
	37	Thiopentone
	38	Succinylcholine

	39	Non-depolarising muscle relaxant
	40	Oxytocin/Ergotamine
	41	Magnesium Sulphate
	42	Phenobarbital / Phenytoin

Essential equipment for emergency and critical care

**Equipment Indicators**	43	Clock with second hand
	44	Gloves - clean
	45	Gloves - sterile
	46	Sharps disposal
	47	Running water & soap
	48	Oral airway (Guedel) – adult & paediatric sizes
	49	Suction machine (foot powered or electric) & tubing
	50	Laryngoscope (working)
	51	Endotracheal Tubes – adult & paediatric sizes
	52	Rigid neck collar or Sandbags/Towel rolls and head restraints
	53	Chest tube & underwater seal (or equivalent)
	54	Pulse oximeter
	55	Bag valve mask (Ambu bag)
	56	Stethoscope
	57	Foetal stethoscope
	58	Blood pressure cuff
	59	IV cannulae – adult size (eg 18G)
	60	IV cannulae – paediatric size (eg 22G, 24G)
	61	IV giving sets
	62	Needles
	63	Syringes – 2ml & 5ml
	64	Urine catheters & bags
	65	Gauze & bandages
	66	Skin disinfectant
	67	Torch
	68	Electricity 24hours/day
	69	Light suitable for clinical examination
	70	Bedside blood sugar testing device & strips
	71	Weighing scales
	72	Thermometer
	73	Refrigerator
	74	Nasogastric Tubes
	75	Oxygen concentrator / cylinder with face masks or nasal prongs and tubing
	76	System for ensuring continuous availability of oxygen (eg reserve electricity generator / reserve cylinders with good transport and refilling system)

Routines for managing emergency and critically ill adults and children

**Routines Indicators**	 77<br>78	System for categorising patients according to clinical urgency (triage)<br>For adults<br>For children
	 79<br>80	System for prioritising the treatment of critically ill patients before stable patients<br>For adults<br>For children
	81<br>82	Admission registration and payment delayed until after triage and emergency treatment<br>For adults

		For children
	83	ICU admission/discharge criteria
	84<br>85	Nurses have a routine of frequent observations of the patients (hourly or specified depending on clinical need)<br>For adults<br>For children
	86<br>87	Clinicians check patients (ward rounds) at least twice a day<br>For adults<br>For children
	88	There is a system for identifying critically ill patients on general wards and transferring to ICU (A "track and trigger" system)

Guidelines for managing emergency and critically ill adults and children

**Guidelines Indicators**	89<br>90	Guidelines for triage<br>For adults<br>For children
	91<br>92	Guidelines for Emergency Care<br>For adults<br>For children
	93<br>94	Guidelines for Critical Care<br>For adults<br>For children
	95	Guidelines for Oxygen use

Support Services for managing emergency and critically ill adults and children

**Support Services Indicators**	96	Lab with facilities and trained personnel to measure Haemoglobin
	97	Lab with facilities and trained personnel to measure blood glucose
	98	Lab with facilities and personnel to measure Serum Urea/Creatinine, Sodium and Potassium
	99	X-ray facilities and trained personnel for chest radiographs
	100	System for emergency blood transfusion
	101	System for making cross matched blood available within 1 hour of blood sample arriving in lab
	102	System for testing donor blood for the viruses HIV, Hepatitis B & C
	103	Lab with facilities and trained personnel to do direct microscopy & bacterial gram stain
	104	Lab with facilities and trained personnel to do bacterial culture and antibiotic sensitivities

IV intravenous; IM intramuscular;

Advanced Emergency & Critical Care Indicators

The hospital has:

1	Ventilator
2	Piperacillin/Meropenem
3	Colloid
4	Fresh Frozen Plasma
5	Propofol or Midazolam
6	Noradrenaline or Dobutamine
7	Invasive Blood Pressure Monitoring
8	Central Venous Pressure Monitoring
9	Aterial Blood Gas analysis
10	Syringe pump

References

1. WHO. Emergency Triage Assessment and Treatment (ETAT). Geneva: WHO Press; 2005.
2. WHO. Guidelines for Essential Trauma Care: WHO Press; 2004
3. WHO. Pocket Book of Hospital Care for Children. Geneva: WHO; 2005.
4. AMDDColumbiaUniversity. Toolbook for Improving Quality of Emergency Obetric Services. 2003.
5. WHO. Surgical care at the district hospital. Geneva: WHO Press; 2003.
6. WHO. Generic Essential Emergency Equipment List. Geneva; 2006.
7. WHO. Anaesthesia at the district hospital. Geneva; 2000.
8. Watters D, Wilson I, Leaver R, Bagshawe A. Care of the Critically Ill Patient in the Tropics. Oxford: Macmillan Publishers; 2004.
9. NICE. Acutely ill patients in hospital: recognition of and response to acute illness in adults in hospital. National Institute for Health and Clinical Excellence Clinical Guideline 50. 2007.
10. NCEPOD. An Acute Problem? Report by The National Confidential Enquiry into Patient Outcome and Death. 2005.
11. EMSSA. Emergency Medicine Society of South Africa Practice Guidelines http://www.emssa.org.za/documents.php (Accessed 6/3/09). 2008.
12. Jarman B, Gault S, Alves B, Hider A, Dolan S, Cook A, et al. Explaining differences in English hospital death rates using routinely collected data. Bmj. 1999; **318**(7197): 1515-20.
